# Do Largest Shareholders Incentively Affect Financial Sustainability Under Holdings Heterogeneity? Regulation/Intermediary of Financial Constraints Through Managerial Behavior Games

**DOI:** 10.3389/fpsyg.2022.754608

**Published:** 2022-02-10

**Authors:** Lipai Zhang

**Affiliations:** School of Business and Management, Shanghai International Studies University, Shanghai, China

**Keywords:** agency cost, real estate industry, managerial behavior, sense of ownership, incentive, supervision effect

## Abstract

The real estate industry is characterized by a high degree of financial intensity and is more significant in certain areas. The relative enterprises require certain financial ability and large shareholders’ controlling power to support their survivals and competitiveness. However, due to the multiple adverse impacts of current state policies on banks and private capital, the problem of capital restraints of real estate has become increasingly serious. From a corporate governance perspective, this paper studies the interactions among financial constraints, ownership concentration and corporate performance under different shareholding states: by analyzing the quantitative characteristics of equity structure and searching for the appropriate range of the largest shareholder holding ratio, which has considered both the financial sustainability and characteristics. It is found that raising the ownership concentration could enhance supervision effect rather than encroachment, effectively ease the financial constraints and improve the performance of enterprises, both of which are significant under high ownership concentration. Financial constraints play a significant intermediary effect in absolute holdings and have obvious regulatory effects in decentralized equity. Also, the mechanisms of ownership concentration are reflected in the strengthening of corporate supervision, reduced agency costs, improved operating efficiency, and increased investment attractiveness. The adjusted behavior adds to the responsibility awareness rather than free-ride psychology, forming a dynamic game on financial decisions. Their financial sustainability in areas would provide a nationwide reference for governance reform and managerial behavior.

## Introduction

As the cornerstone of corporate governance, the ownership structure provides a realistic basis for the allocation of corporate residual control rights and residual claim rights (Wruck, 2014). The shareholding structure reflects the rights to speak and checks and balances of shareholders as the owners in corporate reform (Kahn and Winton, 1998; Deng et al., 2013; Foss et al., 2020). Moreover, it is an important part of the “principle-agent” relationship in the reform of the enterprise system. The equity-structure adjustments of listed enterprises are worldwide. The relevant international cases are focusing on dealing with agency costs. The phenomenon is also increasing in China, such as the “Vanke Equity Change” in 2015 and equity holdings of Evergrande Group reduced in 2021, all have aroused widespread and continuous attention. These events are more frequent in developed areas in China (as the eastern seaboard represents the fastest reform of split share structure), which may even bring unprecedented challenges to corporate governance and even survival. Specifically, ownership structure reform could bring about changes in the organization and personnel of the enterprise in the short term, and finally impact the established managerial structure; its changes would also affect the ability of the enterprise to continue operations in the long term and adjust the “principle-agent” relationship between owners and business operators (Wruck, 2014). The shareholding structure of enterprises has reached relative equilibrium in the “dynamic game” of concentration and checks and balances of multiple shareholders’ shareholdings and has achieved new challenges under external shocks such as market changes and the introduction of new economic policies (Wruck, 2014; Wu et al., 2019). Giving priority to supervision or encroachment requires the allocation of equity (Ahn, 2019; Arif et al., 2021).

Under the existence of market information asymmetry, ordinary enterprises have external financial constraints, and it seems difficult to obtain sufficient credit support. Therefore, their internal existing financial resources are highly dependent. The existence of insufficient investment hinders potential performance improvement; and agency conflicts caused by internal entrust make it difficult to serve the overall interests of the enterprise effectively (Edwards and Pinkerton, 2020). Agents do not always act in the best interests of the principal. For example, the manager acts as the agent of all shareholders, but may not always protect the interests of shareholders, and maximizes his own interests when making decisions. In the principal-agent relationship, due to information asymmetry, the contract between shareholders and managers is incomplete, and the “ethical self-discipline” of the manager needs to be relied on (Wu et al., 2019). In the case of “multiple financial difficulties,” the reform of corporate shareholder equity may provide ways to improve their performance. A reasonable shareholding structure is a prerequisite for the stable development of an enterprise. Prior studies have shown that it has a strong correlation with operating performance – the former determines a relatively high degree of the internal binding force and the manager’s duties, which helps the enterprise operate effectively and improve competitiveness in the market (Slovin and Sushka, 2012; Huang and Chen, 2019; Huang et al., 2019). Namely, it provides institutional mitigation to principal-agent conflict.

Structural adjustment of large shareholders’ equity is regarded as the top priority of equity reform. It determines the rationality of shareholder structure and the right to speak of large shareholders. The degree of ownership concentration could significantly change the way and effect of shareholders’ exercise of rights, form core control force among all shareholders, and further affect the organizational stability, strategic development mode, and governance ability of the enterprise (Edge et al., 2010; Wruck, 2014; Edwards and Pinkerton, 2020).

In China, during the past two decades, enterprises have generally seen phenomena of equity concentration. In the process of over 40-year reform and opening up, China has become the second-largest economy. Facing the competition of world-class multinational corporations, the concentration of large shareholders’ holdings (especially the largest shareholder) is supposed to optimize the investment decisions and performance within a suitable range; however, the previous literature lacks sufficient research on the role of equity trends in different holding states, and the role of financial constraint in the transmission of “Ownership concentration-Corporate performance” lacks in-depth discussion. Based on this, the article will examine the effect of corporate equity structure adjustments by studying “the interactions between ownership concentration trends, financial constraints, and corporate performance.” The impact mechanism of equity changes on performance will be taken from the “supervisory awareness” and “responsibility awareness” of large shareholders, which bring about the changes in their managerial behaviors (Wruck, 2014). The structural reform may have direct or indirect effects on the continuous operation of the enterprise.

Based on the principles of data openness, comparability, and effectiveness, in order to better observe the effect of external financing pressure on corporate equity reform and the impact of equity structure changes on corporate governance, a typical capital-intensive industry – real estate industry is selected as the object of analysis. As an important engine of the national economy, this industry is inseparable from ample financial support. The influence of the real estate industry on the national economy is mainly reflected in the importance of stimulating and stabilizing the economy. First of all, from the perspective of driving the economy, the real estate industry can drive the development of a series of upstream and downstream industries such as steel, building materials, machinery, chemicals, ceramics, textiles, home appliances, and so on. These bring more opportunities in the labor markets. Based on statistics, the real estate industry directly or indirectly affects more than 60 industries. Considering the contribution of real estate development investment to GDP through related industries and consumption, the total contribution of real estate development investment to economic growth currently exceeds 20%. Secondly, from the perspective of economic stability, due to the high degree of industrial correlation, the large fluctuations of real estate development investment can lead to fluctuations in other related industries, which will inevitably lead to economic fluctuations. At the same time, as a large part of the funds of developers and individual home buyers are bank loans, at least more than 40% of the actual investment in real estate development comes from bank loans. Therefore, maintaining the steady growth of real estate development investment is also of great significance for the sustainable and stable development of the national economy.

While since 2013, tightening of credit policies-the promulgation of the new “Five National Principles” has led to a serious reduction in funding for housing projects. The average annual interest rate of real estate loans has increased to the range of 15 to 18%, and the interest rate of private financing exceeds 30%. It is also stricter in banks’ mortgage requirements, thereby suppressing the financial leverage effect. With the triple attack of national policies, capital markets, and banks, most real estate enterprises’ turnover growth has slowed down. The housing mergers have been intensified, and the industry’s concentration has increased. Due to factors such as efficiency and internal control, the trend of ownership concentration is more common in real estate enterprises. Relevant empirical analysis of this industry is helpful to investigate the practical significance of the equity concentration. In addition, according to the characteristics of the real estate industry, another goal is finding the appropriate scope of holdings for large shareholders.

This paper takes real estate enterprises as an example and uses panel data from WIND^1^ China Stock Market Accounting Research (CSMAR) databases to analyze their relationship under different shareholding levels. The research procedures are as follows.

**FIGURE 1:**
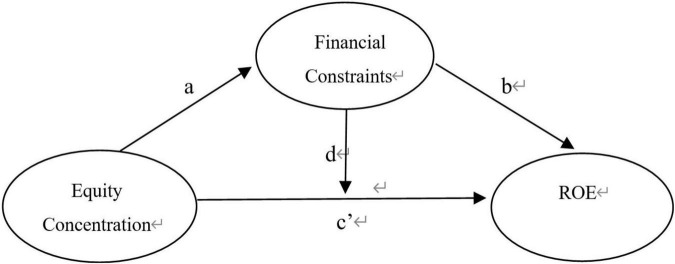
Interactive relationship model of “Equity Structure – Financial constraints – Enterprise Performance.”

Firstly, the regulation and mediation of financial constraints are considered in turn. In previous works of literature, financial constraints were mainly taken as explained variables to consider how to be alleviated. However, easing financial constraints is not always the ultimate goal. A healthy financing situation should serve their business performance. The easing of financial constraints can “unbind” performance expansion and provide the material basis for operation and production. Since few works of literature emphasize its indirect effect, therefore, this paper takes financial constraints as intermediary and regulatory variables, respectively: to explore the mode of action of financial constraints based on the path of “ownership concentration-financial constraints-enterprise performance” and observes the specific utility of financial constraints in different shareholding states. The model constructed is seen in Figure 1.

Secondly, the enterprises are grouped based on diverse holding states, and the utility differences in the sub-sample groups are observed. This paper compares the interaction between ownership concentration and enterprise performance under diverse ownership status and deeply explores the mechanism of ownership concentration. In addition, in order to guide enterprises to improve internal control, the research on the reasonable range of the shareholding ratio of the largest shareholder would provide a reference for the existing corporate system reform, internal control improvement, and sustainable development (Fazzari et al., 2000; Ielpo, 2012).

The conclusions are based on empirical results. In terms of ownership concentration, the proportion of the largest shareholder should be controlled in the range of “20 to 50%” or above 50%, so as to exercise effective control over the enterprises, ensure good efficiency and encourage the large shareholders to participate in the enterprise’s activities more actively. As for the specific range, the business performance, financial issues, management system, and so on should be considered comprehensively. When an enterprise is faced with certain financial constraints, it could change the state of equity decentralization and strengthen equity control, reduce internal friction caused by a power struggle among shareholders and the hidden danger of “excessive control” in the original state, which in turn improve decision-making efficiency to ensure the normal turnover. Under the current financial constraints, real estate enterprises are faced with fierce market competition and have to obtain sustained benefits to support survival. Therefore, according to the empirical results, large shareholders should maintain a medium-high degree of holding and maintain the proportion of the largest shareholder close to or over 50%, so as to reduce the incentive and marginal impact of large shareholders being misappropriated.

## Analysis

In today’s world, ownership concentration is the organizational form of the ownership structure in most enterprises. The change of equity trend often affects two kinds of agency costs directly or indirectly. The first kind of agency cost is a principal-agent contradiction, which mainly arises from the division of labor and asymmetric information (Edwards and Pinkerton, 2020). The second kind of agency cost is reflected between shareholders’ conflict, involving “trench defense effect,” “tunnel effect” and other ways to obtain private interests through deceptions, which requires ownership structure adjustments (Wruck, 2014; Wu et al., 2019; Zhang et al., 2020; Clintworth et al., 2021).

By strengthening the centralization of equity, large shareholders will have more control and attention to enterprises, which could alleviate financial constraints through strengthening supervision, easing credit pressure, and improving investment attraction (Weisbach et al., 2010). Firstly, the supervision effect of large shareholders is conducive to improving the corporate governance mechanism, alleviating the conflicts between shareholders and managers, and making managers serve the goal of maximizing shareholders’ wealth as much as possible. Under the equity incentive, the sense of responsibility and enthusiasm of the largest shareholders could be realized by strengthening the daily management (Wruck, 2014; Wu et al., 2019). Driven by the sense of ownership, these shareholders would bind their personal interests with the interests of the enterprise, which promotes them to improve the capital utilization, reduce the adverse phenomena such as excessive investment, and then reduce the capital pressure (Edwards and Weichenrieder, 2004; Wu et al., 2019). So hypothesis H1a is put forward.

H1a: With other conditions remaining unchanged, financial constraints are inversely proportional to ownership concentration.

However, there are also studies suggesting that the centralization of the largest shareholders is not conducive to enterprise information disclosure. The related information asymmetry will cause both parties to face “moral hazard” after the transaction. Moral hazard is a question raised when studying insurance contracts. Economists often use moral hazard to summarize “lazy,” “free-riding,” and opportunistic behavior (Wruck, 2014). In the governance of listed companies, it usually manifests the following three situations: one is breaching the loan agreement and changing the use of funds privately (Edwards and Pinkerton, 2020); the second is that the borrower conceals the investment income and evades the payment obligation; the third is that the borrower is indifferent to the use of borrowed funds and is not responsible, not working hard, resulting in loss of borrowed funds (Weisbach et al., 2010; Wu et al., 2019).

Information asymmetry could be intensified and even distorted, making outside investors’ investment costs increase. Investors then appeal the extra pay to make up the related risk, which further makes the rising costs of external financing. Enterprises, especially those in small-and-medium size, are deeply sunk into financing dilemmas. In addition, with the increase of large shareholders’ holdings, their behavior of seeking personal gains may also occur, resulting in damage to the enterprise’s assets. Its typical performance is “self-interested merger and acquisition of large shareholders.” This will exacerbate the second type of agency problem (Denis et al., 1997; Cho, 1998; Chin et al., 2009). So H1b is the opposite hypothesis.

H1b: If other conditions remain unchanged, financial constraints are in proportion to ownership concentration.

Similarly, the academic circle has found that when the degree of financial constraint is controlled, the increase of ownership concentration can effectively reduce the first type of agency cost, ensure the consistent motivation of managers’ behavior with maximization of shareholders’ equity, which reduces the probability of managers’ seeking profits for personal gain (Wruck, 2014). At the same time, ownership concentration also promotes the growth of business profits and enterprise value by improving operating efficiency and reputation. At the same time, with the increase of shareholdings, the motivation of large shareholders’ occupation could be gradually offset by the increasingly strong sense of “ownership,’ so it is less possible to “hollowing out” the enterprise value (Jia-Xing, 2007; Slovin and Sushka, 2012; Gyapong et al., 2019). The free-rider effect would also be shrunk. Therefore, hypothesis H2a is proposed.

H2a: Under the condition of existing financial constraints, ownership concentration degree could improve enterprise performance significantly.

Strengthening equity centralization may be accompanied by the phenomenon that large shareholders occupy more equity. Not only in emerging markets, but also among developed countries with perfect civil law, there are cases reflecting the “tunnel effect,” and the Asian financial crisis in 1997–1998 is a typical one. Large shareholders of listed companies will always “dig underground tunnels under the sun” through various means to dig out the wealth of minority shareholders, transfer the assets or profits and empty the listed companies. Tunnels act in many ways (Slovin and Sushka, 2012; Wruck, 2014; Huang and Chen, 2019).

For performance issues, if the concentration of equity slightly decreases, it can form an effective check among shareholders, making enterprise decision-making more “democratic” (Huang and Chen, 2019). The check and balance role among shareholders, could prevent the operation risk caused by their arbitrary or wrong ideas, and supervise the selfish behavior of large shareholders. It can also encourage minority shareholders to participate more actively in daily activities (Edwards and Pinkerton, 2020). The decentralized ownership structure also effectively restrains the “trench defense effect” and “tunnel effect.” If the concentration of equity is reduced, it would be difficult for large shareholders to misappropriate assets and transfer corporate profits, thus maintaining the rational allocation of corporate resources (Dasgupta et al., 2011). Accordingly, hypothesis H2b is proposed.

H2b: Under the condition of existing financial constraints, reducing ownership concentration can significantly improve enterprise performance.

As for the impact of ownership concentration on corporate performance, the current research pays little attention to the potential impact of corporate financial constraints (Slovin and Sushka, 2012; Foss et al., 2020). The influence of ownership concentration on corporate performance can be transmitted in three ways: (1) the improvement of ownership concentration brings about changes in corporate governance, which directly affects performance ability; (2) the improvement of ownership concentration brings about the change of the financial constraint (acting as an intermediary variable), so as to produce the result of “unbinding” or “straitened constraints” for enterprise performance (Slovin and Sushka, 2012); (3) when the improvement of ownership concentration brings about changes in financial performance, financial constraints may play a regulating role. As a moderator variable, enterprises with higher financial constraints may have more obvious “action elasticity” and space to reduce constraints, and the conduction utility may be greater (Weisbach et al., 2010; Liu et al., 2019; Hegde et al., 2020). So we have the following hypothesis.

H3: In the transmission process of ownership concentration degree to enterprise performance, financial constraints have significant regulating and mediating effects.

In China, large-scale real estate enterprises have a large credit base and fewer financing constraints. At the same time, large-scale real estate enterprises do not have the limitation of cash reserves, often have a large amount of cash flow, their non-financial capital limitation, cash sensitivity are far lower than other enterprises. Therefore, large-scale enterprises have a high degree of freedom in cash flow management, and the management may use cash assets for self-interest (Huang and Chen, 2019). This will have a negative impact on the enterprise’s project investment behavior and financial performance. The effect of ownership concentration on financing constraints and corporate performance, as well as the moderating effect of financing constraints, are significantly affected by corporate asset size. Based on the property scale of real estate enterprises, this study proposes hypothesis H4.

H4: Compared with small-scale enterprises, ownership concentration has a relatively low effect on financing constraints, a relatively high effect on corporate performance, and a small regulatory effect on financing constraints.

## Materials, Method, and Design

### Sample Selection and Pre-treatment

This paper attempts to disclose the influence mechanism of ownership concentration and create a reasonable proportion for the largest shareholder. The basic registration information and main financial index data collected in this study were from the WIND (Shanghai Wind Information Technology Co., Ltd., Shanghai, China) and CSMAR (Shenzhen GTA Education Technology Co., Ltd., Shenzhen, China). These collected data belong to the corporate research series in the two mentioned databases. The period is from 2012 to 2018. STATA 16, (StataCorp., College Station, TX, United States) was used for statistical analysis and test.

The investigated samples are limited to the real estate industry for the following reasons: (1) Real estate industry is a highly capital-intensive industry with a long project investment cycle and high risk. If there exist financial constraints, it will directly lead to difficulties such as insufficient investment, operating loss, idle assets, and even the continuous operation threat. Since the promulgation of restrictive policies such as the new “Five National Principles,” real estate enterprises have seen their financing costs rise and their channels become narrower. This is conducive to the observation of financial constraints. (2) Real estate investment has long been regarded as a barometer of China’s economic development, and the comprehensive performance of such enterprises will bring a significant surplus to the national economy. The real estate industry is related to the national economy and people’s livelihood. It provides daily necessities for ordinary people and is the solid foundation of national economic construction. In the process of China’s reform and opening up for more than 40 years, investment centered on the real estate industry has driven economic growth for a long period of time. It also involves many industries, such as iron and steel, concrete, furniture, household appliances, and other important industries. For the vast number of developing countries represented by China, with a huge population, weak industrial carrying base, and obvious advantages of being a latecomer, they need a pillar industry like the real estate industry that can drive the economy on a large scale, especially the economy of backward areas. Therefore, the study of the real estate industry has universal significance. (3) As the foundation of corporate governance, the ownership structure has a strong correlation with corporate performance; However, the concentration of equity in China’s real estate enterprises is more common. In this highly capital-intensive industry, shareholders can effectively control the enterprise’s financial flow and business performance with the increase of the shareholding ratio. A decentralized ownership structure will restrict resource allocation and strategic unity. (4) Over 70% of enterprises in this industry are headquartered in eastern coastal areas, which are across 14 provinces and municipalities in China. They are at the forefront of market reform, and their listed information is more available and transparent. This helps better understand their ownership trends and radiation effect. (5) Real estate industry is being reorganized by the Chinese government and its prospect requires more attention. From “housing not speculation” to “curb the rise of housing prices,” the soul of real estate regulation has changed – the country’s real estate policy from the past economic policy to people’s livelihood and social policy. As credit tightens, there is a risk that many companies will run out of cash next. Real estate has changed, and demand will change over time. In 2021, the Ministry of Housing and Urban-Rural Development and other eight departments jointly issued the Notice on Continuing to Rectify and Standardize the order of the real estate market, and many local governments have followed up. Up to the end of 2021, at least 20 cities, including Tianjin, Xiamen, Zhejiang, Jiangxi, Shanxi, Liaoning, Yunnan, Suzhou, Anhui, Shandong, Hainan, Guangdong, Hebei, Fujian, Heilongjiang, and Xinjiang have issued action plans to regulate the order of the real estate market, according to incomplete statistics from China Real Estate Website, which forms a threat to the industrial ownership and daily business. From the rectification action plan announced around the point of view, real estate development, housing sales, housing leasing, property services in four aspects of the outstanding problems will be the focus of the rectification direction, as these are rooted in agency problems. In recent years, the real estate industry chaos caused the central government to level high concerns. To rectify business and strengthen control from within the industry, it is necessary to clarify and solve the increasingly prominent contradiction between principals and agents through equity allocations. (6) There is a dominant phenomenon in the ownership structure of the real estate industry. According to the descriptive statistical results, the largest shareholder is generally in a relative holding state, and close to the level of absolute holding. All the largest shareholders have the right to participate in the day-to-day management decisions of the company. Most of them share the major rights and have a potential enhancement in equities. If they could share more equities, the effect of managerial behavior may have an essential difference on corporate decisions and governance. In terms of ownership structure, the characteristics of real estate are worth analyzing and verifying more than any other industry (Hartzell et al., 2019).

A total of 868 data sets of 124 A-share enterprises listed on the Shanghai and Shenzhen Stock Exchanges for seven consecutive years from 2012 to 2018 were selected as the original research samples. Samples collected are rejected according to the following criteria: (1) enterprises with missing or discontinuous material data and abnormal index values; (2) insolvent enterprises; (3) cross-listed enterprises within and outside China.

To eliminate the influence of the extreme value of a continuous variable, the outlier Winsor shrinkage was performed (before and after 1 and 99% quartile, respectively) to make them equal to corresponding quantile values.

### Variable Setting and Model Construction

#### Variable Settings

The variables selected are shown in Table 1.

**TABLE 1:** Variable construction.

Variable types	Variable	Symbol	Calculation and description
Explanatory variables	Corporate financial performance	*ROE*	Net profit per year/total final net asset
Explanatory variables	Financial constraints	*SA*	SA index = −0.737*Size+ 0.043*Size^2^−0.04*Age
	Ownership concentration	*OC*	The proportion of the largest shareholder
Control variables	Degree of equity balance	*OB*	The percentage of the top 10 shareholders/the percentage of the largest shareholder −1
	Establishment of fixed number of year	*Age*	The number of years which is logarithmically treated
	Cash on hand	*CH*	The enterprise’s annual cash capital/final total assets
	The capital structure	*RAL*	Total ending liabilities/total final assets
	Asset turnover capacity	*TAT*	Total asset turnover, calculated by current operating income/total assets at the end of the period
	Fixed assets ratio	*FAR*	Fixed assets/total assets at the end of the period
	Enterprise growth ability	*Tobin Q*	Market value of the enterprise/replacement cost of assets
	Net operating cash flow	*NCF*	Net operating cash flow/total assets at the end of the period
Dummy variable	Year fixed effect	*Year*	Dummy variable: if the financial data belongs to a certain year from 2012 to 2018, the value of that year is 1; otherwise, it is 0
	Nature of equity fixed effect	*EN*	Dummy variable: it can be divided into 7 categories according to their nature: private, central or local state-owned holding, provincial state-owned holding, collective, foreign capital and others. EN is 1 if it belongs to a certain class, otherwise, it is 0

##### Financial Constraints (*SA*)

In recent years, most scholars use “Investment-Cash flow sensitivity,” “*KZ* index” and “credit rating” to measure the degree of financial constraint. However, because this method contains many indicators when the randomness of sample data is strong, the results will not be accurate enough. It is evident in the case of policy changes, enterprises entering a new growth cycle, and strategic adjustment. The above indicators will change significantly, making the same indicators not comparable in different stages. Therefore, the *SA* index is selected to measure financial constraints.

Size is represented by the value of the total book value of assets; Age is the cumulative years from the incorporation or merger to the current condition.

##### Corporate Performance (Return on Equity)

From the perspective of shareholders, it should consider the maximization of shareholder benefit. The return on equity (*ROE*) is used as the main measure and the return on assets (*ROA*) is used for the robustness test.

##### Ownership Concentration

Ownership concentration *(OC)* reflects the concentration degree of the enterprises’ shares among large shareholders. The indicators mainly include the proportion of the largest shareholder (TOP 1), the proportion of the top five shareholders (TOP 5), and the proportion of the top ten shareholders (TOP 10). In most cases, the level of corporate ownership concentration is mainly measured by the proportion of the largest shareholder. When the largest shareholder holds more than 50% of the shares, he or she has absolute control; if the shareholding ratio is between 20 and 50%, the enterprise is in a relatively concentrated holding state; if the largest shareholder holds less than 20% of the shares, the enterprise is in shareholding dispersion state.

**FIGURE 2:**
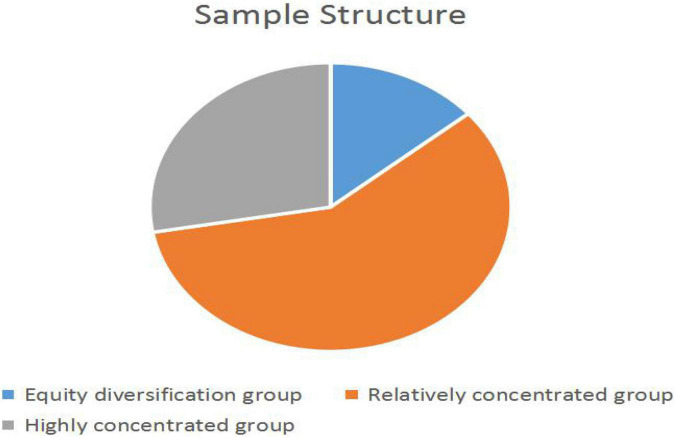
The sample structure of real estate enterprises based on equity.

In China, the ownership concentration of the real estate industry is relatively high. From 2012 to 2019, 56.22% of China’s real estate enterprises were in the state of relative holding and 27.79% were in the state of absolute holding. At the same time, the ratio between the shareholding ratio of the largest shareholder and the sum of the shareholding ratio of the next nine large shareholders is 4.5495 on average. This could be seen in Figure 2.

Therefore, it can be seen that the largest shareholder plays an important and even dominant role in the internal control and operation of the enterprise. Based on this, the shareholding ratio of the largest shareholder is used to measure the level of ownership concentration.

##### Control Variable Group

① *EB* measures the equity checks and balances based on “the ratio of top ten shareholders to the largest shareholder −1.” This indicator pays attention to the influence of the remaining nine shareholders on the largest shareholder.

② The number of years (*Age*) of the enterprise is a common control variable, reflects the development of the enterprise, business foundation, and the ability to resist financial risks to a certain extent; the cash holding set (*CH*) and net cash flow from operating activities (*NCF*) reflect the daily liquidity of the enterprise since their abundance could reduce the external financing pressure and alleviate the shortage of investment. Liability-asset ratio (*RAL*), total asset turnover ratio (*TAT*), fixed asset ratio (*FAR*), and enterprise growth ratio (*TobinQ*) are commonly used business indicators, which, respectively, measure the enterprise’s solvency, capital operation ability, production equipment input status, and sustainable development ability. They are conventional indicators of performance in multi-dimensional situations.

##### Dummy Variables

① This paper firstly controls the year fixed effect to overcome the disturbance factors which may influence the study due to the time change.

② Secondly, because the research samples are limited to the real estate industry in China, the industry effect has been controlled. In view of China’s special institutional environment, state-owned enterprises are often limited by strict operating rules, and the “selfish” behavior of agents is often severely restricted and punished. Therefore, the agency cost and the risk of “vacancy” or “offside” are usually low. And private enterprises often appear “the first type of agency problem” in the economic transition period. Therefore, this paper will control the differences in the nature of the controlling equity.

#### Model Constructions

Based on the research hypothesis, models (1) and (2) are established to test hypotheses 1 and 2:


(1) $$SA_{i,t}=a_{0}+a_{1}OC_{i,t}+\text{φ}control_{i,t}+u_{i,t}$$

(2) $$ROE_{i,t}={\text{γ}}_{0}+{\text{γ}}_{1}OC_{i,t}+{\text{γ}}_{2}SA_{i,t}+\text{φ}control_{i,t}+u_{i,t}$$

For hypothesis 3, the mediating effect and the regulating effect of financial constraints are tested and subgroup regression is performed.

## Empirical Results and Discussion

### Descriptive Statistics

In the whole sample group (as is seen in Table 2), the mean *ROE* and its standard deviation are 0.092 and 0.111, respectively, indicating that during the sample period, real estate enterprises have a positive return on assets but with certain fluctuations. The mean value and standard deviation of *SA* are 5.303 and 1.912, respectively (the variation is relatively large). *OC* has little volatility, with an *SD* of 0.162 and a mean value of 0.392, indicating relatively concentrated ownership. For equity checks and balances, the average proportion of the largest shareholder is 1.657 times that of the following nine large shareholders, which means that the largest shareholder has clear control over the enterprise.

**TABLE 2:** Descriptive statistical results of the whole sample.

Variable	Obs	Mean	Std. dev.	Min	Max
*ROE*	849	0.092	0.111	−0.744	0.65
*SA*	849	5.303	1.912	0.876	10.558
*OC*	849	0.392	0.162	0.1	0.796
*OB*	849	0.657	0.678	0.03	3.081
*Age*	849	3.259	0.153	2.895	3.587
*NCF*	849	0.01	0.096	−0.268	0.271
*RAL*	849	0.646	0.176	0.125	0.92
*TAT*	849	0.268	0.161	0.02	0.95
*Tobin Q*	849	1.671	1.452	0.84	11.69
*FAR*	849	0.037	0.065	0	0.422
*CH*	849	0.145	0.093	0.021	0.528

From the control variable group, real estate enterprises generally have a certain number of years since establishment, among which the minimum value after logarithmic treatment is 2.895 (that is, 16.44 years). There are insufficient net operating cash flow (mean value is only 0.01), a high operating debt ratio (mean value is 0.646), and insufficient turnover capacity (mean value is merely 0.268). However, the current growth indicators present a good average of 1.671, reaching the peak of 11.69, which will encourage enterprises to continue increasing investment spendings.

For the description of the key indicators, it could be divided into equity diversification group, relatively- concentrated group, and highly concentrated group according to the situation of ownership concentration, so as to compare whether there is a difference between the key indicators.

As shown in Table 3, when the equity of an enterprise is relatively dispersed, the average return on assets (*ROA*) is 5.8%, significantly lower than the industry average. Compared with the relatively concentrated group and the highly concentrated group, this group is with a gap of 2.7 and 6.6%, respectively. At the same time, its mean *SA* is the lowest, that is, the degree of financial constraint is the highest. In the ownership concentration index, the largest shareholder holds most of the shares, and the mean value of the three sample groups is in stepped form – 15.9, 35.1, and 59.3%. In *Tobin Q*, an indicator representing growth, the mean value of the equity dispersion group is 2.598, much higher than that of the other two groups (1.574 and 1.417, respectively). Therefore, it is preliminarily concluded that there may be a positive relationship between ownership concentration, financial constraint, and operating performance.

**TABLE 3:** Descriptive statistical results of key indicators in the sub-sample group.

Variable	Obs	Mean	Std. dev.	Min	Max
**State of equity dispersion: shareholding ratio of the largest shareholder is less than 20% (sub-sample)**					
*ROE*	117	0.058	0.077	−0.154	0.328
*SA*	117	4.523	2.274	0.876	10.558
*OC*	117	0.159	0.032	0.1	0.2
*Tobin Q*	117	2.598	2.932	0.84	11.69
**Relative concentration of equity: the shareholding ratio of the largest shareholder is between 20 and 50% (sub-sample)**					
*ROE*	496	0.085	0.118	−0.744	0.65
*SA*	496	5.292	1.906	0.876	10.558
*OC*	496	0.351	0.09	0.2	0.5
*Tobin Q*	496	1.574	1.12	0.84	11.69
**Highly concentrated equity: the largest shareholder holds more than 50% shares (sub-sample)**					
*ROE*	236	0.124	0.102	−0.366	0.514
*SA*	236	5.713	1.587	2.982	10.003
*OC*	236	0.593	0.08	0.5	0.796
*Tobin Q*	236	1.417	0.457	0.84	3.37

As is shown in Table 4, the interpretation of *SA* and *OC* financing restrictions is closely related to *ROE* performance, with correlation coefficients of 0.301 and 0.200, respectively. At the same time, the control variable groups reflecting the financial capability of enterprises are also significantly correlated with *ROE*, which proves the effectiveness of the selection of control variable indicators.

**TABLE 4:** Correlation results of variables.

Variables	*(1)*	*(2)*	*(3)*	*(4)*	*(5)*	*(6)*	*(7)*	*(8)*	*(9)*	*(10)*	*(11)*
*(1) ROE*	1.000										
*(2) SA*	0.301*	1.000									
*(3) OC*	0.200*	0.186*	1.000								
*(4) OB*	−0.018	0.092*	−0.653*	1.000							
*(5) Age*	0.107*	−0.055	−0.133*	0.129*	1.000						
*(6) NCF*	0.102*	−0.059	−0.005	−0.002	0.004	1.000					
*(7) RFAL*	0.169*	0.616*	0.196*	0.005	0.001	−0.093*	1.000				
*(8) TAT*	0.178*	−0.130*	−0.027	−0.013	−0.031	0.235*	−0.003	1.000			
*(9) Tobin Q*	−0.155*	−0.503*	−0.182*	0.146*	0.094*	−0.011	−0.404*	−0.003	1.000		
*(10) FAR*	−0.111*	−0.195*	−0.033	−0.133*	−0.089*	0.087*	−0.122*	0.261*	0.049	1.000	
*(11) CH*	0.117*	−0.239*	0.060	−0.070*	0.067	0.251*	−0.346*	0.144*	0.248*	−0.077*	1.000

* correlation is significant at the 5% probability level.

Ownership concentration *(OC)*, *OB*, and *SA* are strongly correlated, indicating that adjusting the holding status may play a role in alleviating financing constraints. At the same time, we found that the OC variable was significantly correlated with *Age*, *RFAL*, *Tobin Q*, etc., thus proving the significance of the adjustment of ownership structure and corporate finance.

Among the control variables, the correlation between *RFAL* and *Tobin Q* and *SA* is 0.616 and −0.503, respectively (both significant at the probability level of 5%), indicating a strong correlation between capital structure and growth and financing constraints. In the analysis of the above table, the absolute values of correlation coefficients are all less than 0.5, thus overcoming the multicollinearity problem.

### Multiple Regression Results

#### Regression Results-Assumption 1 and 2

In hypothesis 1, financial constraint *SA* is seen as the explained variable. After main regression, it is divided into (1) Equity diversification group (no more than 20%), according to the proportion of shares held by the largest shareholder; (2) Relatively concentrated group (between 20 and 50%); (3) Highly-concentrated (over 50%). In Hypothesis 2, *ROE* is taken as the explained variable, and regression is performed according to the same grouping standard as hypothesis 1. Table 5 shows the results.

**TABLE 5:** Models (1) and (2): multiple regression results.

Variables	*SA*	*SA* (<0.2)	*SA* (0.2–0.5)	*SA* (>0.5)	*ROE*	*ROE* (<0.2)	*ROE* (0.2–0.5)	*ROE* (>0.5)
*SA*					0.024***	0.015***	0.026***	0.034***
					(6.04)	(4.64)	(5.02)	(4.07)
*OC*	2.503***	2.635	3.374**	3.077*	0.120***	0.216	0.048	0.144**
	(4.08)	(0.37)	(2.54)	(1.77)	(3.70)	(0.92)	(0.63)	(2.51)
*OB*	0.669***	0.274	0.747***	0.508	0.008	0.013	0.001	0.010
	(4.27)	(1.00)	(3.25)	(0.38)	(0.89)	(1.27)	(0.09)	(0.19)
*Control Variables*	Control	Control	Control	Control	Control	Control	Control	Control
*Constant*	4.352*	−10.16	6.448**	5.315*	−0.431***	−0.249**	−0.554***	−0.423**
	(1.87)	(−1.26)	(2.32)	(1.97)	(−4.04)	(−2.48)	(−3.74)	(−2.03)
Firm	Control	Control	Control	Control	Control	Control	Control	Control
Industry	Control	Control	Control	Control	Control	Control	Control	Control
Year	Control	Control	Control	Control	Control	Control	Control	Control
Observations	849	117	496	236	849	117	496	236
*R*-squared	0.588	0.758	0.600	0.583	0.275	0.551	0.262	0.335
*F*	25.96	15.06	15.85	14.24	10.65	5.56	8.30	4.87
*P*-value	0.000	0.000	0.000	0.000	0.000	0.000	0.000	0.000

In brackets are t statistics, ***P < 0.01, **P < 0.05, *P < 0.1; standard errors clustering at state-level in parentheses.

The regression of hypothesis 1 shows that the *SA* index can be significantly increased by strengthening the ownership concentration (at the 1% probability level). Namely, the financial constraints can be effectively reduced, and the corresponding coefficient value is 2.503. This empirical result is corresponding to the prior conclusions. In the sub-sample regression, the utility in the decentralized equity group was not significant; while that in the relatively centralized equity group and the highly centralized equity group were significant under the probability of 5 and 10%, respectively, and the corresponding coefficient values were 3.374 and 3.077, respectively. In the state of equity dispersion, the motivation and effect of marginal encroachment of the largest shareholder are generally more obvious, which will result in interest infringements and resource deprivations to other shareholders, thus restricting the positive effect of increasing ownership concentration. However, in the sample group where large shareholders occupy an important or even dominant position, their self-interested behavior and marginal motivation are relatively insufficient, so that the positive utility of increasing ownership concentration is greater than the negative utility, thus effectively reducing financial constraints. The essence of the change of equity trend is the dynamic game between the “selfish” behavior and the “overall interests of the enterprise” under the goal of maximizing the shareholders’ wealth.

Real estate enterprises in this industry seem to face more financial allocation issues with high-level market homogenization pressure. The relative concentration of equity has a good easing effect on the financial constraints of enterprises, which is conducive to raising capital, expanding operations, and creating more income. In particular, the real estate industry is such a capital-intensive industry, so that the state should attach great importance to its negative impact caused by financing restrictions.

Compared with the enterprises in dispersed equity, the willingness to pay cash dividends is usually higher in the shareholder-controlled enterprises, which can effectively alleviate the capital constraints of real estate institutions and obtain more support from investors. The enterprises in developed provinces are usually under more market attention, thus strengthening ownership holdings should be more effective. If the largest shareholder holds a larger share, the supervision effect can be more effective to reduce the “first-type agency cost,” trigger the “eyeball-attraction effect” of enterprises to attract financing, relieve the situation of capital strain, and promote the maximization of shareholders’ interests.

Through the regressions examine hypothesis 2, it is found that an increase in the *SA* index (i.e., a reduction in financial constraints) could improve *ROE*, both significantly at the 1% level. In the main regression, increasing the ownership concentration would significantly improve *ROE*, and the corresponding coefficient value is 0.12. However, in the sub-sample regressions, the coefficient of *OC* on *ROE* was not obvious in both the relatively concentrated group and the equity dispersion group. Only in the highly concentrated equity group could *ROE* be effectively improved, with a probability level of 5%. In the case of non-absolute control, the positive effects of increased supervision, increased operating efficiency, and reduced agency costs brought about by the increase in the shareholding of large shareholders are still offset by the “trench defense effect” and “tunneling effect”; in the state of absolute control, the encroachment effect of large shareholders is reduced, and the stewardship effect under the “ownership consciousness” could be brought into play, thereby significantly improving operating performance.

In sum, if the share of the largest shareholder is low, according to the “trench defense effect,” the increasing shareholding will significantly enhance the motivation and behavior of the minority shareholders to seize the interests and inhibit the benign progress of the enterprise. When the largest shareholder is in the absolute controlling position, the higher control right can promote the managers to meet the shareholders’ goals and improve productivity performance to the greatest extent under the existing mechanism.

#### Multiple Regression Results-Assumption 3

As for the role of financial constraints between “ownership concentration” and “enterprise performance,” the mediating effect and the regulating effect are considered, respectively.

##### Mediating Effect

As is shown in Figure 1, after controlling other variables, we set the coefficient of equity concentration on financing constraints as ‘a’; considering the variable of equity concentration, the coefficient of financing constraints on corporate performance is ‘b’; when the financing constraint *SA* is not controlled, the effect of equity concentration on enterprise performance is ‘c’; after controlling the *SA* situation of financing constraints, the effective coefficient of equity concentration on enterprise performance is ‘c’.’ According to the holding rates of the largest shareholder, it is grouped into three conditions, as shown in Table 6.

**TABLE 6:** Regression results of mediating effect.

Mediating effect assessment	Total effect (c)	Direct effect (c′)	Indirect effect (ab)	A	b	Note
**Total state of equity (full sample)**						
Coefficient β	0.181	0.120	0.06	2.50	0.024	The indirect effect coefficient column is the product of the estimated values of A and B, whose significance depends on the significance of A and B.
*T*-value	4.95	3.70	/	4.08	6.05	
Significance	0.000	0.000	/	0.000	0.000	
**State of equity dispersion: shareholding ratio of the largest shareholder is less than 20% (sub-sample)**						
Coefficient β	0.256	0.216	0.040	2.635	0.015	If the total effect C is not significant, the analysis of mediating effect will be stopped.
*T*-value	0.91	0.92	/	0.37	4.64	
Significance	0.370	0.369	/	0.718	0.000	
**The relative concentration of equity: the shareholding ratio of the largest shareholder is between 20 and 50% (sub-sample)**						
Coefficient β	0.136	0.048	0.088	3.373	0.026	If the total effect C is not significant, the analysis of mediating effect will be stopped.
*T*-value	1.64	0.63	/	2.54	5.02	
Significance	0.104	0.532	/	0.013	0.000	
**Highly concentrated equity: the largest shareholder holds more than 50% shares (sub-sample)**						
Coefficient β	0.250	0.144	0.105	3.078	0.034	
*T*-value	2.64	1.78	/	1.77	4.07	
Significance	0.011	0.081	/	0.082	0.000	

In the total sample, the intermediary effect of financial constraints is significant, and the proportion in the total impact is 0.06/0.181 = 0.33. However, the effect is not significant in the decentralized group and the relatively centralized group. In the highly concentrated equity group, the impact of financial constraints is significant, and the proportion of total impact is 0.105/0.250 = 0.42. It is seen that under the premise of absolute holding, the intermediation effect of financial constraints is highly significant.

Under the absolute controlling status of “I am the majority,” the increase of ownership concentration will improve the control power of large shareholders over the management and give full play to the “supervision effect” rather than the “expropriation effect.”

As the overall interests of the enterprise are gradually consistent with those of large shareholders, these shareholders will attach more importance to financial distribution and organizational management, so as to enhance cash holdings, curb financial constraints, and realize “shareholder wealth maximization.”

##### Regulatory Effect

Tables 7, 8 examine the moderating effect.

**TABLE 7:** Regulatory effect test results – based on R2.

Model	*R* ^2^	Adjusted_R2	Variation of R2	Variation of *F*	Change in significance of *F*
1	0.275	0.256	0.275	0.275	0.000
2	0.279	0.259	0.004	4.492	0.034

**TABLE 8:** Regulatory effect test results – based on multiple regression results.

Variables	ROE_Z	ROE_Z	ROE_Z (<0.2)	ROE_Z (0.2–0.5)	ROE_Z (>0.5)
*SA_Z*	0.420***	0.421***	1.150**	0.461***	0.483**
	(9.102)	(9.140)	(2.24)	(6.81)	(2.12)
*OC_Z*	0.175***	0.184***	0.486	0.098	0.193
	(4.102)	(4.296)	(0.191)	(0.97)	(1.33)
SA_Z* OC_Z		0.073**	0.581**	0.101	0.082
		(2.119)	(2.21)	(1.09)	(0.52)
*OB_Z*	0.051	0.069	0.061	0.032	0.064
	(1.158)	(1.556)	(1.17)	(0.42)	(0.19)
*Control Variables*	Control	Control	Control	Control	Control
*Constant*	−0.097	−0.134	0.692	0.223	−0.511
	(−1.060)	(−1.447)	(1.13)	(1.05)	(−1.34)
Firm	Control	Control	Control	Control	Control
Industry	Control	Control	Control	Control	Control
Year	Control	Control	Control	Control	Control
Observations	849	849	117	496	236
*R*-squared	0.256	0.259	0.464	0.2285	0.2634
*F*	10.647	13.441	5.56	7.37	4.65
*P*-value	0.000	0.000	0.000	0.000	0.000

In brackets are t statistics, ***P < 0.01, **P < 0.05, *P < 0.1; standard errors clustering at state-level reported in parentheses.

To avoid the multicollinearity problem, all data indicators were centralized, and then the two models were regressed, respectively. Model 1 and Model 2 both take enterprise performance as the explained variable, and the explanatory variable and control variable remains unchanged. However, Model 2 adds the interaction item of *SA* and *OC* after standardization. It can be seen from Table 8 that the change in R square is 0.004, and its significant change in F is 0.034, which is highly significant under the probability of 5%, thus confirming the existence of the regulatory effect.

In Table 7, it can be seen from sub-samples that, after the addition of interaction terms, the interpretation strength of the equation becomes stronger, increasing from 0.256 to 0.259; the coefficient on the interaction term is positive, which is significant at 5% probability, indicating that the mitigation of financial constraints could play a positive regulatory role, and it is mainly significant in the equity dispersion group.

According to Table 8, the financial constraint of the equity dispersed group is much greater than that of the other two groups, and it has more obvious “action elasticity” and reduced constraint space. If it is alleviated, the conduction utility may be greater.

The interaction between the financial constraint index (*SA*) and the ownership concentration degree (*OC*) can play a positive regulatory role. That is, the higher the degree of financial constraint, the lower the conduction effect. Meanwhile, it plays a significant role in the equity diversification group. In the state of relatively concentrated and highly concentrated groups, enterprises tend to have good execution efficiency, making financial constraints on its regulatory effect relatively small; in the case of decentralization of equity, minority shareholders are the main body, which plays a large role in restraining among shareholders, so the implementation and supervision effect of large shareholders is weak.

#### Multiple Regression Results-Assumption 4

According to the scale of enterprise assets, this paper divides them into three equal parts. As can be seen from Table 9, compared with small-scale enterprises, the effect of ownership concentration on financing constraint is relatively weak (the effective coefficient of OC of medium and large enterprises is not significant), but relatively high (the effective coefficient of OC of medium and small enterprises is not significant).

**TABLE 9:** Multiple regressions based on different firm size.

Variables	SA (Small-size)	SA (Medium-size)	SA (Large-size)	ROE (Small-size)	ROE (Medium-size)	ROE (Large-size)
SA				0.022*	0.024	0.022***
				(1.70)	(1.60)	(3.41)
OC	1.202***	0.415	−0.238	0.082	0.100	0.163*
	(3.26)	(1.33)	(−0.28)	(1.31)	(1.59)	(1.86)
OB	0.290**	0.110	0.078	0.009	0.017	0.009
	(2.34)	(1.17)	(0.40)	(0.56)	(1.05)	(0.46)
Age	−1.230***	−0.503*	−0.488	0.099**	0.129**	0.069
	(−3.61)	(−1.73)	(−0.33)	(2.16)	(2.48)	(1.09)
NCF	0.205	−0.151	−0.226	0.189**	−0.017	−0.078
	(0.57)	(−0.48)	(−0.25)	(2.62)	(−0.36)	(−0.77)
RFAL	0.506**	0.787**	1.263	0.003	−0.142	0.013
	(2.24)	(2.31)	(1.13)	(0.10)	(−1.33)	(0.15)
TAT	−0.853	−0.515*	0.007	0.101**	0.187***	0.262***
	(−1.06)	(−1.81)	(0.01)	(2.65)	(3.33)	(2.85)
Tobin Q	−0.314***	−0.206*	−0.117	−0.003	0.022	0.009
	(−10.28)	(−1.93)	(−0.49)	(−0.35)	(1.41)	(0.37)
FAR	−0.853	−0.399	−2.704	−0.191**	−0.287	0.024
	(−1.06)	(−0.49)	(−0.68)	(−2.12)	(−1.21)	(0.10)
CH	−0.151	−0.372	−0.752	0.151**	0.127*	0.202
	(−0.35)	(−1.03)	(−0.39)	(2.02)	(1.80)	(1.61)
Constant	7.729***	6.831***	9.637*	−0.420**	−0.688***	−0.472**
	(6.83)	(7.77)	(1.71)	(−2.47)	(−4.03)	(−2.07)
Firm	Control	Control	Control	Control	Control	Firm
Industry	Control	Control	Control	Control	Control	Industry
Year	Control	Control	Control	Control	Control	Year
Observations	282	283	284	282	283	284
*R*-squared	0.7163	0.2888	0.3096	0.2901	0.2877	0.2783
*F*	31.26	5.05	4.57	4.58	4.77	7.35
*P*-value	0.000	0.000	0.000	0.000	0.000	0.000

In brackets are t statistics, ***P < 0.01, **P < 0.05, *P < 0.1; standard errors clustering at state-level reported in parentheses.

#### Robustness Check

Table 10 shows the robustness check.

**TABLE 10:** Robustness test results.

Variables	*SA*①	*SA*②	*SA*③	*ROA*①	*ROA*②	*ROA*③	*ROA_Z*	*ROA_Z*
*OC*	2.574***	0.841***	1.883*	0.043***	0.0435*	0.0477**	0.452***	0.251*
*SA*				0.006***	0.007***	0.006***	0.162***	0.413**
SA*OC							0.078**	0.099*
*Control variables*	Control	Control	Control	Control	Control	Control	Control	Control
Firm	Control	Control	Control	Control	Control	Control	Control	Control
Industry	Control	Control	Control	Control	Control	Control	Control	Control
Year	Control	Control	Control	Control	Control	Control	Control	Control
Observations	849	849	732	849	236	849	849	117
*P*-value	0.000	0.000	0.000	0.000	0.000	0.000	0.000	0.000

In brackets are t statistics, ***P < 0.01, **P < 0.05, *P < 0.1; standard errors clustering at state-level reported in parentheses.

In the test of the relationship between financial constraints and ownership concentration: considering the limitations of the selected growth indicators (which cannot accurately reflect the market price information, and have price deviation), the control variable *Tobin Q* was changed into dynamic price-earnings ratio (*PE*). Namely the ratio between equity price and annual after-tax profit per share. After winsorize, tail reduction, the coefficient of *OC* was 2.574 and the *P*-value was 0, which were still proportional and highly significant. On this basis, the fixed effect of individual enterprises was added to the equations, and the *OC* coefficient was still positive and highly significant. Based on these, the samples are limited to the relatively concentrated group and the highly concentrated group, because these two groups are significant in the empirical study of the relationship between them. The test results show that the coefficient is 1.883, which is significant under 10% probability.

On the premise of controlling financial constraints, when studying the relationship between ownership concentration and enterprise performance: multi-dimensional analysis is used to change the indicators of corporate performance from *ROE* to *ROA*, and the control variable *Tobin Q* is further changed into dynamic price-earnings ratio (*PE*), which is applied to the regression detection of interactive relations. Results in the positive relationship test between ownership concentration and financial performance, *OC* beta coefficient was 0.043 and the *P*-value was 0. Then the sample was limited to the highly concentrated equity group (which was the only significant group in the empirical test above), and the *OC* coefficient was 0.0435, which was significant under the probability of 10%. Above these, a new control variable – the logarithm of the annual capital value of the enterprise is added. Since the enterprise performance is often closely related to its scale, this index is used to control the number of enterprise resources and carrying capacity, and the regression result is still significantly under the probability of 5%.

In the mediating effect test for financial constraint (replace *ROE* with *ROA*), the corresponding coefficients on ‘a’ and ‘b’ are highly significant and positive, and the indirect influence is also significant. The total effect coefficient value is 0.0572 and significant at 1% probability, so the two have a positive mediating effect. In the adjustment effect test, the enterprise performance index was changed to *ROA*, and the sample range of enterprises was controlled to be the total group and the equity dispersed group, respectively. The interaction coefficient of *SA* and *OC* was 0.078 and 0.099, which were significant and verified the effectiveness of the adjustment effect.

The above results are consistent with the above results. Therefore, the research results are persuasive.

#### Mechanisms of Ownership Concentration

The goals pursued by shareholders and managers are inconsistent. Shareholders want to maximize the value of the equity they hold, while managers want to maximize their own utility. Therefore, there is a moral hazard between shareholders and managers, which needs to be guided by incentive and restraint mechanisms. This is also the principle of equity incentives.

Based on previous studies, the structural equation model (*SEM*) was used to explore the influence mechanism of ownership concentration on corporate performance: ownership concentration has a direct and indirect influence on corporate performance. Among them, the indirect path includes “free-rider behavior” and “operational efficiency,” which successively reflect the “responsibility consciousness” and “supervision consciousness” of large shareholders.

The fitting values of Tables 11, 12 were obtained by AMOS 24 software (SPSS, Chicago, IL, United States) and the maximum likelihood method. At the same time, the probability *P*-value of chi-square statistics is 0.71 (a fitting standard greater than 0.05), that is, under the probability of 5%, the Chi-square statistics are not significant, and there is no significant difference between the sample data and the theoretical model. For these set potential variables: (1) total asset turnover (*TAT*) and operating income growth rate (*GRI*) for “operating Efficiency,” this path is simplified as *OE* (Operational Efficiency); (2) “Free Ride” is expressed by the number of senior executives (*CEO*) and the size of the board of directors, and the action path is simplified as *FR* (free-ride). The selected variables are used as the mediating variables for regression analysis, and the variable group of the original equation remains unchanged.

**TABLE 11:** Structural equation model (SEM) estimation results.

Effect of path	coefficient	Standard error	Critical ratio	*P*-value	Standardized coefficient
*OE←OC*	0.246	0.024	10.12	0.000	0.067
*ROE←OE*	0.018	0.002	8.47	0.000	0.062
*FR←OC*	−0.159	0.017	−9.39	0.000	−0.067
*ROE←FR*	0.002	0.003	2.68	0.011	0.005
*ROE←OC*	0.027	0.007	3.68	0.000	0.025

**TABLE 12:** Path decomposition of the equity trend of the largest shareholder affecting corporate performance.

Equity adjustment trend	Paths types	Influence paths	Influential effect	Contribution degree	Relative rate
Ownership concentration	Direct	*OC→ROE*	0.027	0.027	85.05%
	Indirect	*OC→FR→ROE*	−0.159 * 0.002 = −0.000318	0.000318	1.00%
		*OC→OE→ROE*	0.246 * 0.018 = 0.004428	0.004428	13.95%
		Sum	0.00411	0.004746	14.95%
	Total		0.03111	0.031746	100%

*The contribution degree is the absolute influence of a certain path on the performance under the trend of concentration of shareholders’ equity. It does not distinguish the direction.*

According to the results in Tables 10, 11, the path coefficient of the direct effect of ownership concentration on performance is 0.027, and this effect is significant at 1% of the probability, with the contribution reaching 85.05%. Indirect effects mainly function through “inhibiting free-rider effect” and “improving operation efficiency,” indicating that there are some mediating effects reaching a total of 14.95%.

According to the regression results: (1) from the perspective of corporate governance, ownership concentration can strengthen the power and enterprise management of the controlling shareholders. It is reflected in simplifying and optimizing the organizational structure, which makes the management perform their duties more seriously and reduces free-rider behavior. As can be seen from the regression coefficient in Table 10, the increase of ownership concentration can significantly reduce the size of *CEO* and board of directors, inhibit the psychology of “free rider,” and enhance the sense of responsibility of management, but the mediating effect contribution rate is only 1%. (2) Secondly, from the perspective of operational efficiency, under the trend of ownership concentration, large shareholders who are driven by the “sense of supervision,” would pay more attention to the operation and construction of enterprises. This phenomenon would strengthen the professional labor division and process optimization, improve the efficiency of income generation of assets. The contribution rate of this effect is 13.95%, which plays a major role in the mediating effect. Thus, it can be seen that “supervision consciousness” will be the main factor for the improvement of enterprise performance.

The real estate agent should change the cost of the institution under the condition of “dispersed equity,” increase the proportion of large shareholders appropriately, and change the proportion into “relatively- concentrated” or “highly concentrated” equity. Also, enterprises should give full play to the “supervision effect” and “incentive effect” of large shareholders.

## Conclusion and Suggestions

The innovation of this study is to compare the effect of equity structure adjustments on real estate enterprises under different shareholding states, as well as the intermediary/regulatory effect of financial constraints in the transmission process, and to explore the influence mechanism of ownership concentration inside enterprises on operation and financing. According to the characteristics of the real estate industry in China, it is supposed to consider the reasonable shareholding range of the largest shareholder, so as to provide a useful reference for the academic circle.

### Conclusion

#### Financial Constraints and Ownership Concentration Are Negatively Correlated

If other conditions remain unchanged, the financial constraint is inversely proportional to the ownership concentration degree. The increase of ownership concentration degree will effectively alleviate the financial constraint. At the same time, it is found that this relationship is significant in the group with relatively- concentrated and highly concentrated ownership, but not significant in the group with dispersed ownership. We are consistent with Marco and Röell (1998), Nguyen et al. (2015).

#### Concentration of Equity Is Positively Correlated With Enterprise Performance

Under the condition of fixed financial constraints, the increase of ownership concentration can significantly improve enterprise performance. But in the sub-sample regression, only ownership concentrated groups are obvious.

#### In the Transmission Process of Ownership Concentration to Corporate Performance, Financial Constraints Have Significant Regulatory and Mediating Effects

The intermediation effect of financial constraint is mainly reflected in the state of the absolute holding state. The increase of ownership concentration will effectively alleviate financial constraints, “loosen” and “reduce pressure” for enterprise investment/production activities. When the ownership concentration is not high enough, the mediating effect under financial constraints is difficult to emerge due to the inefficiency caused by the “trench defense effect,” “tunnel effect” and “first type agency problem.”

#### There Are Some Differences in the Interaction Between Ownership Concentration, Financing Constraints, and Firm Performance Due to Firm Size

In large-scale enterprises, compared with small-scale enterprises, ownership concentration has a relatively insignificant effect on financing constraints, but a relatively significant effect on corporate performance. However, the moderating effect of financing constraints does not show a significant difference between scales. Large-scale enterprises have a high degree of freedom in cash management and risk of management’s “selfish” behavior. Strengthening ownership concentration can effectively restrain managers and limit the influence of their self-interested behavior on corporate finance. This supplements research on Roggi and Giannozzi (2015), Safiullah and Shamsuddin (2018).

### Suggestions on the Real Estate Industry

#### Moderately Increase Ownership Concentration to Improve Performance

As a capital-intensive industry, the real estate industry should attach great importance to financing constraints in order to achieve sustainable survival and development. The real estate enterprises should change the problem of agency cost under the state of “equity dispersion,” moderately increase the proportion of large shareholders, and change to the state of “relatively concentrated” or “highly concentrated” equity; Give full play to the “supervision effect” and “incentive effect” of large shareholders to improve operating efficiency, reduce agency costs and improve financial performance.

At the level of ownership concentration, the shareholding proportion of the largest shareholder should be controlled at “20–50%” or higher than 50%, so as to effectively control the enterprise and the management. As for the specific range, the enterprise’s operating performance, financing constraints, management system, and so on should be considered comprehensively.

#### To Avoid “Hollowing Out by Large Shareholders” and Protect the Interests of Minority Shareholders

At the same time, real estate enterprises should avoid the “tunnel effect” and “trench defense effect.” Within a certain proportion of shares, the motive of large shareholders’ embezzlement becomes stronger with the increase of their shares. The corresponding management mechanism should be improved to form certain checks and balances on the largest shareholder, avoid the encroachment of large shareholders as far as possible, limit their irrational behavior of cash dividends, and ensure the legitimate interests of minority shareholders and play a role in corporate governance.

The government should also help to set a good ownership structure, improve the disclosure of real estate listed companies’ information, prevent enterprises from fighting for control, which is not conducive to financing due to internal friction, enhance coordination, better respond to market changes, and create more profits (Lo Storto, 2018).

This paper is limited to data availability and sample size, so does not consider whether corporate financing constraints. Business performance will be affected by the development stage (growth and maturity) of real estate enterprises. Therefore, the next step is to bring the enterprise growth stage and enterprise type into the research category, which enhances literature of Wruck and Wu (2009).

## Data Availability Statement

The raw data supporting the conclusions of this article will be made available by the author, without undue reservation.

## Ethics Statement

Ethical review and approval was not required for the study on human participants in accordance with the local legislation and institutional requirements. The patients/participants provided their written informed consent to participate in this study. Written informed consent was obtained from the individual(s) for the publication of any potentially identifiable images or data included in this article.

## Author Contributions

The author confirms being the sole contributor of this work and has approved it for publication.

## Conflict of Interest

The author declares that the research was conducted in the absence of any commercial or financial relationships that could be construed as a potential conflict of interest.

## Publisher’s Note

All claims expressed in this article are solely those of the authors and do not necessarily represent those of their affiliated organizations, or those of the publisher, the editors and the reviewers. Any product that may be evaluated in this article, or claim that may be made by its manufacturer, is not guaranteed or endorsed by the publisher.

## Footnotes

1 WIND Information is a leading financial data service provider in China and has built a first-class large-scale financial engineering and financial data warehouse centered on financial securities data. WIND database is one of the core service products of Shanghai Wind Information Technology Co., Ltd.

## References

[B1] Ahn Y. (2019). Distress cost and corporate financing policy: evidence from equity options. *Appl. Econ.* 51 4299–4312.

[B2] Arif M. Nazir M. S. Qamar M. A. J. Abid A. (2021). Project finance and recourse loans: determining debt choices in political, economic and financial risk positions under global perspective. *Eur. J. Int. Manag.* 1:1.

[B3] Chin C. L. Chen Y. J. Hsieh T. J. (2009). International diversification, ownership structure, legal origin, and earnings management: evidence from taiwan. *J. Account. Audit. Finance* 24 233–262.

[B4] Cho M. H. (1998). Ownership structure, investment, and the corporate value: an empirical analysis. *J. Financial Econ.* 47 103–121. 10.1016/s0304-405x(97)00039-1

[B5] Clintworth M. Lyridis D. Boulougouris E. (2021). Financial risk assessment in shipping: a holistic machine learning based methodology. *Maritime Econ. Logistics* 1–32. 10.1057/s41278-020-00183-2

[B6] Dasgupta S. Noe T. H. Wang Z. (2011). Where did all the dollars go? The effect of cash flows on capital and asset structure. *J. Financial Quantitat. Anal.* 46 1259–1294. 10.1017/s0022109011000512

[B7] Deng Z. Hofman P. S. Newman A. (2013). Ownership concentration and product innovation in chinese private smes. *Asia Pacific J. Manag.* 30 717–734. 10.1007/s10490-012-9301-0

[B8] Denis D. J. Denis D. K. Sarin A. (1997). Ownership structure and top executive turnover. *J. Financial Econ.* 45 193–221. 10.1016/s0304-405x(97)00016-0

[B9] Edge R. M. Kiley M. T. Laforte J. P. (2010). A comparison of forecast performance between federal reserve staff forecasts, simple reduced-form models, and a dsge model. *J. Appl. Econ.* 25 720–754. 10.1002/jae.1175

[B10] Edwards D. N. Pinkerton E. (2020). Priced out of ownership: quota leasing impacts on the financial performance of owner-operators. *Mar. Pol.* 111 103718.1–103718.10.

[B11] Edwards J. S. S. Weichenrieder A. J. (2004). Ownership concentration and share valuation. *German Econ. Rev.* 5 143–171. 10.1111/j.1465-6485.2004.00100.x

[B12] Fazzari S. M. Glenn H. R. Petersen B. C. (2000). Investment-cash flow sensitivities are useful: a comment on kaplan and zingales. *Q. J. Econ.* 115 695–705.

[B13] Foss N. J. Klein P. G. Lien L. B. Zellweger T. Zenger T. (2020). Ownership competence. *Strategic Manag. J.* 42 302–328.

[B14] Gyapong E. Ahmed A. Ntim C. G. Nadeem M. (2019). Board gender diversity and dividend policy in Australian listed firms: the effect of ownership concentration. *Asia Pacific J. Manag.* 38 603–643.

[B15] Hartzell D. J. Howton S. D. Howton S. Scheick B. (2019). Financial flexibility and at-the-market (ATM) equity offerings: evidence from real estate investment trusts. *Real Estate Economics* 47, 595–636.

[B16] Hegde S. Seth R. Vishwanatha S. R. (2020). Ownership concentration and stock returns: evidence from family firms in India. *Pacific-Basin Finance J.* 61:101330. 10.1016/j.pacfin.2020.101330

[B17] Huang J. Z. Shi Z. Zhou H. (2019). Specification analysis of structural credit risk models. *Rev. Finance* 24 45–98.

[B18] Huang R. H. Chen J. (2019). The rise of hostile takeovers and defensive measures in China: comparative and empirical perspectives. *Eur. Bus. Organ. Law Rev.* 20 363–398.

[B19] Ielpo F. (2012). Equity, credit and the business cycle. *Appl. Financial Econ.* 22 939–954.

[B20] Jia-Xing Y. (2007). Pyramid structure, tax effort of local government, and controlling shareholder’s embezzlement of funds. *J. Manag. Sci.* 20 89–96.

[B21] Kahn C. Winton A. (1998). Ownership structure, speculation, and shareholder intervention. *J. Finance* 53 99–129. 10.1111/0022-1082.45483

[B22] Liu J. Wu Y. Ye Q. Zhang D. (2019). Do seasoned offerings improve the performance of issuing firms? Evidence from China. *Int. Rev. Financial Anal.* 62 104–123.

[B23] Lo Storto C. (2018). Ownership structure and the technical, cost, and revenue efficiency of Italian airports. *Utilities Policy* 50 175–193. 10.1016/j.jup.2018.01.003

[B24] Marco P. Röell A. (1998). The choice of stock ownership structure: agency costs, monitoring, and the decision to go public. *Q. J. Econ.* 113 187–225.

[B25] Nguyen T. Locke S. Reddy K. (2015). Ownership concentration and corporate performance from a dynamic perspective: does national governance quality matter? *Int. Rev. Financial Anal.* 41 148–161.

[B26] Roggi O. Giannozzi A. (2015). Fair value disclosure, liquidity risk and stock returns. *J. Banking Finance* 58 327–342.

[B27] Safiullah M. D. Shamsuddin A. (2018). Risk in islamic banking and corporate governance. *Pacific-Basin Finance J.* 47 129–149.

[B28] Slovin M. B. Sushka M. E. (2012). Ownership concentration, corporate control activity, and firm value: evidence from the death of inside blockholders. *J. Finance* 48 1293–1321.

[B29] Weisbach M. S. Almeida H. Campello M. (2010). The cash flow sensitivity of cash. *J. Finance* 59 1777–1804.

[B30] Wruck K. H. (2014). Equity ownership concentration and firm value: evidence from private equity financings. *J. Financial Econ.* 23 3–28.

[B31] Wruck K. H. Wu Y. L. (2009). Relationships, corporate governance, and performance: evidence from private placements of common stock. *J. Corporate Finance* 15 30–47.

[B32] Wu R. Jiang Z. Shi H. (2019). Love conditionally: the ownership structure and bribery behavior of chinese firms. *Rev. Dev. Econ.* 23 1027–1049.

[B33] Zhang L. Yu W. Xia X. (2020). The interaction between the largest shareholder and firm performance under the heterogeneity of holding-regulation and intermediary effect of financing constraints. *Preprints* 2020:2020100182. 10.20944/preprints202010.0182.v1 32283112

